# First Year Implementation and Performance of the Republic of Korea’s National Antimicrobial Stewardship Program Pilot: Policy Directions for Sustainable Expansion

**DOI:** 10.56786/PHWR.2026.19.43.2

**Published:** 2026-11-12

**Authors:** Young-Il Jeong, Yeo Won Jin, In Kyoung Kim, U Jin Cho, Eun Young Choi, Na-Ri Shin

**Affiliations:** Division of Antimicrobial Resistance Control, Department of Healthcare Safety and Immunization, Korea Disease Control and Prevention Agency Cheongju Korea

**Keywords:** Antimicrobial stewardship, Antimicrobial resistance, Antibiotic consumption, Health policy

## Abstract

**Objectives:**

This report analyzes the implementation and performance of the first year of the Republic of Korea’s National Antimicrobial Stewardship Program (ASP) pilot and proposes policy directions for its sustainable operation and a phased expansion.

**Methods:**

We examined implementation data from 78 participating general and tertiary hospitals with having more than 300 beds from November 1, 2024, to December 31, 2025. Data collected through and annual reports, program guidelines, annual evaluations, and on-site inspection were reviewed to assess ASP infrastructure and major structural and process indicators.

**Results:**

In all, 78 (45.9%) of 170 eligible institutions participated. These institutions created multidisciplinary ASP teams that included 695 personnel and developed 622 institution-specific antibiotic use guidelines. These personnel implemented an average of 5.7 of 7 prescribing intervention activities, with a mean acceptance rate of 81.7%. Restricted antibiotics that included 68 active ingredients were managed, with a mean institutional approval rate of 89.5%. Information systems that supported ASP activities were operated in 73 institutions (93.6%). All of the institutions met the basic evaluation criteria, and 64 (82.1%) received an A or B grade. The total financial support in the first year was KRW 65.97 billion.

**Conclusions:**

The first-year pilot established the ASP workforce, operational systems, and implementation infrastructure through national financial support and evaluation, exhibiting a high level of implementation. However, short-term changes in antibiotic use and antimicrobial resistance were difficult to assess. Longitudinal evaluation, tailored support reflecting workforce and infrastructure gaps, and the gradual transition to outcome-oriented evaluation are needed to support expansion to general hospitals and hospital-level institutions.

**Key messages**

**① What is known previously?**

Before the pilot began, Antimicrobial Stewardship Program in Korean healthcare institutions largely depended on individual institutional efforts, with limited standardized systems and substantial gaps in the workforce, information systems, and financial support.

**② What new information is presented?**

In the first year of the national Antimicrobial Steward­ship Program (ASP) pilot, 78 institutions established multidisciplinary teams including 695 personnel and developed 622 treatment guidelines antibiotics. Institutions implemented an average of 5.7 intervention activities, with an average acceptance rate of 81.7%. ASP information systems were adopted by 93.6% of participating institutions, and 82.1% achieved an A or B grade.

**③ What are implications?**

National financial support and evaluation can strengthen ASP infrastructure and implementation. Tailored support and outcome-oriented evaluation are needed, with their policy effects verified and sustainable expansion guided using longitudinal assessment.

## Introduction

Antibiotic resistance is a major public health problem that threatens the effectiveness of infection treatment and healthcare safety. Antibiotic consumption and resistance rates of major pathogens in the Republic of Korea (ROK) remain high globally, even when considering the unique healthcare environment. In 2024, human antibiotic consumption stood at 33.6 DID^1)^ (defined daily dose per 1,000 inhabitants per day), which was approximately 1.7 times the OECD average of 19.7 DID, showing an increasing trend after 2021 [1-2]. In addition, the proportion of Access antibiotics, which have a relatively low risk of resistance development, accounted for only 38.8%, indicating a need to improve not only antibiotic consumption but also prescribing appropriateness [3]. Regarding resistance rates, the rates of methicillin-resistant *Staphylococcus aureus* and multidrug-resistant *Acinetobacter baumannii* remained at high levels in 2023 at 45.2% and 73.7%, respectively, while the resistance rate of carbapenem-resistant *Enterobacteriaceae* showed a continuous increasing trend. Surveillance results in 2024 also showed that resistance rates of major pathogens generally remained high. In particular, the resistance rate of carbapenem-resistant *Klebsiella pneumoniae* has increased rapidly since 2016, and resistance rates in long-term care hospitals were confirmed to be higher than in other healthcare institutions [4].

The World Health Organization (WHO) recommends active Antimicrobial Stewardship Program (ASP) activities in healthcare institutions as a core strategy to reduce adverse antibiotic events and unnecessary use, improve therapeutic effectiveness, and suppress the emergence and spread of antibiotic-resistant bacteria [5]. To perform effective ASP, healthcare institutions should establish antibiotic use guidelines, implement prior approval for restricted antibiotics, prospective prescription review and feedback, and optimization of duration, dose, and route of administration, based on executive support and the establishment of a responsible dedicated team. Furthermore, systematic operation is required to continuously monitor antibiotic consumption and resistance rates, alongside feedback of results and healthcare personnel education [6].

In the meantime, antimicrobial stewardship activities in domestic healthcare institutions have relied on voluntary efforts by individual institutions, resulting in insufficient establishment of systematic operating structures and standardized management criteria. Additionally, securing specialized and dedicated personnel, such as infectious disease specialists and infectious disease specialist pharmacists, was limited, and information systems and evaluation frameworks for systematically monitoring and giving feedback on antibiotic prescriptions and consumption were also inadequate. Consequently, there were significant variations in the level of ASP activities among institutions, and there were many constraints on conducting continuous and systematic antibiotic management activities [7-10]. Accordingly, the Korea Disease Control and Prevention Agency (KDCA), together with the Ministry of Health and Welfare, Health Insurance Review and Assessment Service, National Health Insurance Service, and related academic societies and experts, prepared an operational plan for the national health insurance-funded ASP pilot project, obtained final approval through the 17th Health Insurance Policy Deliberation Committee on September 4, 2024, and came to operate the government-led ASP pilot project for a total of 3 years and 2 months from November 2024 to December 2027 [7].

This report aimed to analyze the operational status and performance of the first year of the government-led ASP pilot project and to present policy directions for the continuous operation of the project in the future and the step-by-step expansion to medical institutions.

## Methods

In this study, a comprehensive analysis was conducted on the project details performed from November 1, 2024 to December 31, 2025, targeting 78 medical institutions at or above the general hospital level with more than 300 beds that participated in the first-year pilot project. In particular, active use was made not only of the interim and annual result reports submitted by each medical institution but also of project guidelines, annual result evaluations, and on-site inspection data.

## Results

### 1. Overview of the Antimicrobial Stewardship Program Pilot Project

The operational purpose of this pilot project is to induce the appropriate prescription and management of antibiotics by establishing an ASP operational foundation and a regular implementation system within medical institutions (Figure 1). The policy significance of this project lies in its serving as a turning point from a voluntary ASP activity system of medical institutions prior to the project to a systematic national project based on national policy and financial support, through the organic linkage of policy-management evaluation-financial support-field execution.

The pilot project was designed to set minimum essential staffing criteria according to bed capacity and to configure a multidisciplinary dedicated ASP team and decision-making body for each medical institution. Participating institutions must faithfully perform key activities, such as establishing antibiotic prescribing guidelines, prescription interventions, restricted antibiotic approvals, monitoring antibiotic consumption and resistance rates, education and promotion, and building computerized systems. Furthermore, support funds are differentiated by grade from A to D by evaluating bed capacity and the execution results of ASP activities (Tables 1, 2). Target medical institutions eligible to participate in the project were prioritized starting from general hospital levels with more than 300 beds, where the urgency of antimicrobial resistance management and the risk of resistance are high due to high broad-spectrum antibiotic consumption and a large number of inpatients and severe cases.

### 2. Participating Medical Institutions and Specialized Personnel in the First Year

Among the 170 medical institutions eligible to participate in the project, 78 medical institutions (45.9%) located in 16 provinces and cities, excluding Jeju, participated in the project, confirming high interest and policy demand in the clinical field, despite it being the first year of the project. Among participating institutions, those located in the capital region (Seoul, Incheon, Gyeonggi, and Gangwon) showed the highest participation rate at 64.1% (50/78 institutions). By institutional type, 40 out of 47 tertiary general hospitals nationwide (85.1%) and 38 out of 123 general hospitals (30.9%) participated. In particular, the inclusion of most tertiary general hospitals responsible for severe and high-difficulty care into the national ASP management system is evaluated as an encouraging factor for the step-by-step expansion of ASP in the future (Figure 2).

A total of 695 medical personnel participated in this project, forming dedicated ASP teams with an average of 8.9 personnel per institution. Among them, essential personnel totaled 465, including 317 physicians and 148 pharmacists (including pharmacist support staff), and multidisciplinary specialized personnel, comprising specialists in nursing, clinical microbiology, information technology, and administration, totaled 230 across 78 institutions. In particular, among the participating physicians, infectious disease specialists numbered 234 (73.8%), with more than 6 out of 10 infectious disease specialists in the ROK participating (64.1%), thereby securing high ASP expertise despite the first year of the project. Eighteen infectious disease specialist pharmacists participated across 14 institutions, accounting for 12.2% of the total pharmacist workforce. Given that there were only 33 infectious disease specialist pharmacists nationwide at the time of the project, 54.5% of this group participated in the project (Table 3).

### 3. Operation of the Antibiotic Use Management Committee and Establishment of Antibiotic Use Guidelines

To successfully establish an operational ASP system within an institution, ASP governance characterized by a virtuous cycle of performance management, feedback, and improvement must be established based on responsible operation and support through a decision-making system centered on key management. During the first year of the project, all institutions secured momentum for promoting internal ASP by forming and operating Antibiotic Use Management Committees with key management, such as hospital directors, serving as chairpersons. The average number of committee members per institution was 16.7, laying the foundation for multidisciplinary collaboration by combining medical staff from various clinical departments associated with antibiotic prescribing (an average of 8 clinical specialty departments per institution) alongside executives from nursing, pharmacy, and administrative departments (Figure 3). As a result, a sustainable institution-level ASP execution system was established through regular committee operations involving executive management and multidisciplinary participation, which is a core ASP strategy.

Along with committee operations, ASP standards applicable to actual clinical settings must be established by preparing disease-specific antibiotic use guidelines that reflect the clinical environment and microbiological characteristics of each medical institution. To this end, participating institutions established a total of 622 tailored, appropriate antibiotic prescribing criteria (an average of 8 types per institution) reflecting institutional characteristics based on guidelines published by academic societies, KDCA, and WHO, thereby enhancing clinical applicability for various infectious diseases (Table 4). Although there were disparities among institutions, ranging from a minimum of 1 type (5 institutions) to a maximum of 43 types (1 institution), establishing a foundation to advance into a practical management system usable for actual ASP interventions beyond merely presenting simple prescribing criteria through consistent antibiotic prescribing algorithms represents an encouraging achievement of this project.

### 4. Establishing a Virtuous Cycle System for Systematic Antimicrobial Stewardship Management

Antibiotic prescribing intervention is a core activity of ASP that reduces unnecessary or inappropriate antibiotic use and prevents the occurrence of antibiotic resistance by systematically reviewing clinicians’ antibiotic prescriptions and guiding them toward appropriate prescribing. Through this approach, an effective intervention implementation effect that leads to actual prescription changes, along with a reduction in antibiotic consumption, must be realized in the clinical field. In this project, various prescribing intervention activities must be performed according to the conditions of each institution, focusing on prospective audit and feedback.

The seven intervention items presented in the project guidelines include combined antibiotic prescription interventions that adjust duplicate prescriptions of antibiotics with identical agents or overlapping antibacterial spectra, long-term administration interventions that recommend optimal treatment duration, IV-to-oral switch interventions for injectable antibiotics to prevent adverse drug reactions and block infection, antibiotic de-escalation therapy interventions that recommend precise antibiotic use based on microbiological test results and patient status, microbiology test–based antibiotic prescribing interventions that guide the use of the most appropriate antibiotic for the causative pathogen based on microbiological culture and antibiotic susceptibility test results, guideline-adherent antibiotic prescribing interventions, and therapeutic drug monitoring for specific antibiotics that interprets a patient’s serum drug concentration in real time to present the optimal dose and dosing interval for each patient. During the first year of the project, participating institutions actively implemented an average of 5.7 intervention items out of the seven intervention items (Table 4).

In particular, the average ASP intervention acceptance rate^2)^ accepted by prescribers relative to the total number of interventions was 81.7%, confirming that interventions by the dedicated ASP team can lead to prescription changes in actual clinical practice (Table 4). By intervention item, therapeutic drug monitoring for specific antibiotics showed the highest implementation rate at 71 institutions, whereas de-escalation therapy was relatively low at 52 institutions. In addition, the implementation rate by intervention item at tertiary general hospitals was generally higher than that at general hospitals; in particular, a large difference was observed between institutional types in guideline-adherent antibiotic prescribing, confirming the need for support considering the personnel and infrastructure of each institution. In some institutions, concrete performance cases such as a decrease in total antibiotic consumption as well as broad-spectrum and restricted antibiotic consumption were also confirmed after the project. At this point, it is difficult to judge these cases as an effect of the overall project; however, these results demonstrate that ASP can impact actual antibiotic prescribing behaviors and consumption changes beyond a simple management system, suggesting the need to systematically verify the effectiveness of ASP by continuously evaluating qualitative outcomes such as ASP intervention acceptance rates, prescription changes, and discontinuations in the future.

Restricted antibiotic approval management is a core ASP activity aimed at guiding appropriate antibiotic selection for patients and suppressing the occurrence of antibiotic resistance by proactively managing unnecessary or inappropriate use of broad-spectrum antibiotics and antibiotics with a high risk of resistance. In this pilot project, restricted antibiotics were designated considering the characteristics of medical institutions and the risk of antibiotic resistance, and a pre-prescription approval system was established and operated. Participating institutions managed 68 agents across 26 classes, primarily focusing on carbapenems and other antibiotics. Of the 571,197 applications for approval to use restricted antibiotics, 515,109 were approved, resulting in an overall approval rate of 90.2%. The average approval rate by institution was 89.5%, with a median of 91.4%, showing a variation of 42.9–100% across institutions (Table 4). These differences can be influenced by factors such as the timing of ASP introduction, the degree of operational establishment, the awareness of prescribing physicians, and the level of internal collaboration within the institution. Therefore, continuous monitoring by institutions is more important than simple comparisons between institutions. In particular, improvements in prescribing practices were confirmed in some institutions through the active operation of the approval system, such as reductions in the approval rate for restricted antibiotics^3)^ and antibiotic consumption. Cases of restricted antibiotic approval management showed a 14.8–16.7% decrease in approval rates, and some institutions also saw reductions in the consumption of meropenem, vancomycin, and teicoplanin. These results demonstrate the potential for a pre-management system for restricted antibiotics to translate into actual improvements in antibiotic prescribing and usage practices, highlighting the need to evaluate the long-term effectiveness of ASP through continuous monitoring of antibiotic consumption and prescribing practices.

In addition, through the establishment of a standardized resistant bacteria and *Clostridioides difficile* infection (CDI) incidence surveillance system under this pilot project, a framework capable of linked analysis with major outcome indicators, such as antibiotic consumption and intervention activities, was prepared, and a foundation for preparing future policy evidence for key management was secured by identifying organism species with a high burden of resistance and institutional characteristics.

### 5. Antimicrobial Stewardship Program Educational Activities to Change Prescribing Practices of Medical Staff and Improve Awareness of Antibiotic Resistance

The purpose of ASP education and promotional activities is to promote medical staff adherence to antibiotic use guidelines and appropriate prescribing, and to raise awareness of antibiotic resistance and ASP among executive management and staff, thereby establishing a foundation for the sustainable operation of ASP within medical institutions. In this pilot project, education on antibiotic use guidelines targeting prescribing physicians was conducted in parallel with education to improve ASP awareness for staff and executive management, thereby encouraging institution-wide participation and collaboration. Guideline education was conducted at all participating institutions, with an average of 5.3 educational sessions conducted per institution. In addition, awareness education for staff and executive management was conducted at all institutions, occurring at an average frequency of 6.9 sessions per institution, which was higher than that of guideline education for prescribing physicians (Table 4). Promotional activities, which were not a mandatory requirement for project implementation, were also voluntarily conducted at 63 institutions (80.8%), with an average of 3.6 activities per institution, confirming efforts to spread awareness of ASP both within and outside the institutions (Table 4). In particular, at some institutions, education for prescribers and satisfaction surveys conducted after guideline development helped identify and address barriers to intervention. As a result, the average acceptance rate across seven intervention items increased from 57.5% to 92.9%. Furthermore, at institutions where hospital directors directly participated in ASP education, the average ASP intervention acceptance rate was high at 94%. These results demonstrate that ASP education and promotion can go beyond simple information delivery to contribute to improving prescribing behavior and intervention acceptance among medical staff.

### 6. Evaluation of Participating Healthcare Institutions and Support Funds

As a result of the evaluation of participating institutions in the first year, all institutions met the basic requirements, and 64 institutions (82.1%) were evaluated as grade A or B, confirming an overall high level of project implementation and willingness to participate (Table 4). In particular, 86.7% of institutions with 301–600 beds obtained grade A or B without any grade D, indicating that active ASP activities were conducted despite relatively limited infrastructure. On the other hand, grade differences among institutions mainly occurred at the level of securing specialized personnel, such as infectious disease specialists and pharmacists. This finding suggests that, for the stable operation of ASP, customized support considering institutional personnel and infrastructure conditions is necessary, along with policy support to strengthen the securing of specialized personnel in the future. The total amount of support funds for participating institutions in the first year reflected in the evaluation results was 65.97 billion KRW, with approximately 24.6 billion KRW in advance support funds and approximately 41.4 billion KRW in retrospective support funds disbursed (Table 4).

## Conclusion

Through the national government-led healthcare institution ASP support policy based on the health insurance fee schedule, establishing a systematic management system in healthcare institutions that previously faced difficulties in operating ASP independently due to personnel and financial constraints, improving the quality of antibiotic use, ensuring patient safety, and establishing a national management foundation to suppress the occurrence and spread of antimicrobial resistance can be considered major first-year achievements of this pilot project.

Through the first-year project, participating institutions met the basic indicators and established an ongoing ASP operational framework centered on committee operation, guideline formulation, prescription intervention, surveillance, and education, demonstrating a high level of project implementation and potential for field settlement. In particular, the participation of specialized personnel, including infectious disease specialists, and multidisciplinary personnel contributed to establishing a prescription management system based on expertise and collaboration, and the 64.1% participation rate of infectious disease specialists is evaluated as a major achievement in the initial introduction and settlement of ASP. In the future, infectious disease specialists are expected to play a pivotal role in expanding ASP through collaborative networks between leading and adopting institutions. In addition, pharmacists were identified as key personnel responsible for conducting ASP interventions, confirming their important role. Continued training and development of specialist pharmacists are needed in preparation for program expansion.

In terms of intervention activities, various intervention items were introduced and implemented in a short period, and the institutional average ASP intervention acceptance rate was 81.7%, confirming that the intervention activities of dedicated ASP teams are being accepted in actual clinical settings. However, the acceptance rate for some items, such as interventions on combined (duplicate) antibiotic prescriptions, was relatively low at 62.5%, indicating the need for management support and strengthened multidisciplinary collaboration to increase intervention effectiveness. Furthermore, by establishing a continuous surveillance system for antibiotic consumption, resistance rates, and CDI incidence, a foundation was secured to comprehensively evaluate the correlation among future antibiotic prescribing, consumption, and resistance changes.

As mentioned above, this project is meaningful in that it secured a foundation for future continuous management of appropriate antibiotic use and program expansion by establishing a structural foundation for ASP targeting domestic medical institutions to introduce and implement various intervention activities according to institutional conditions. Although achievements such as a reduction in total antibiotic and broad-spectrum/restricted antibiotic consumption and an increase in ASP intervention acceptance rates following education were confirmed in some participating institutions, it is difficult to generalize them as common effects across all participating institutions. Therefore, in the future, it is necessary to analyze the effects of intervention activities from multiple perspectives according to the characteristics of medical institutions and ASP operation levels by utilizing the accumulated data during the 3-year pilot project period. Specifically, it is necessary to compare and evaluate intervention volume, acceptance rates, and changes in antibiotic prescribing according to medical institution types, such as tertiary general hospitals and general hospitals; presence of infectious disease departments and participation of infectious disease specialists; and the size, composition, and input level of dedicated personnel. In addition, it is necessary to evaluate the contribution of computer systems to ASP operational efficiency and intervention performance by analyzing the types of construction and operation of computer systems by medical institution and the level of utilization of key functions, such as prescription surveillance, intervention support, and automatic calculation of consumption. Furthermore, longitudinal changes in antibiotic consumption and resistance rates of major resistant pathogens should be tracked, and medium- to long-term effects following ASP implementation should be objectively validated through comparative analysis considering institutional baseline characteristics and pre-participation levels.

Ultimately, these analysis results can be utilized to identify differences in ASP operation performance and key influencing factors by medical institution and to establish evidence-based project operation models, including effective personnel allocation and computer system utilization strategies. Furthermore, to ensure the sustainable operation of ASP in domestic medical institutions and its phased expansion to all general hospitals, efforts should be made to transition the pilot project into a full-scale program. Differentiated support should be provided based on the size of each medical institution and its levels of specialized personnel and infrastructure. In addition, a collaborative system centered on leading institutions should be established to support the early adoption of ASP in medical institutions with limited experience in its implementation.

## Figures and Tables

**Figure 1:**
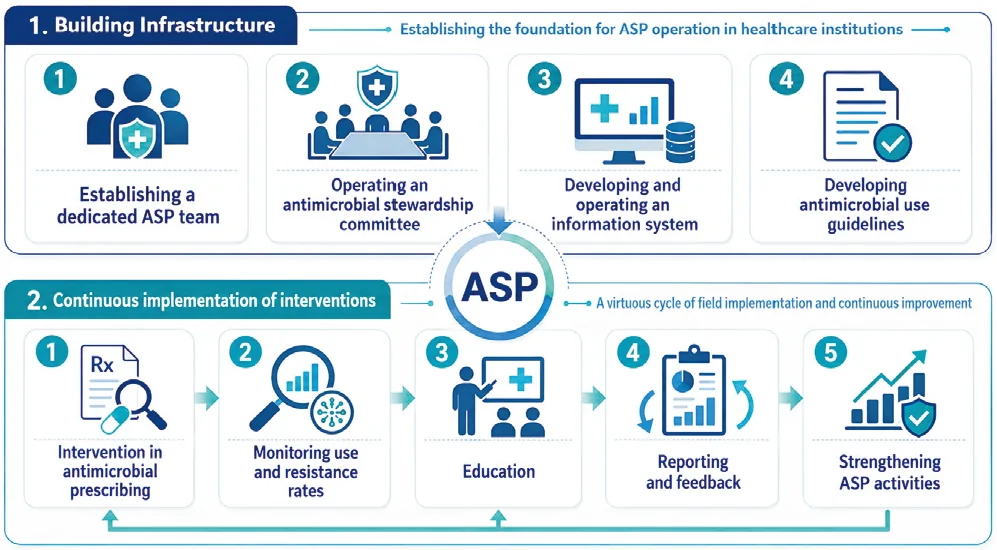
ASP Infrastructure and the ongoing implementation framework ASP=Antimicrobial Stewardship Program.

**Figure 2:**
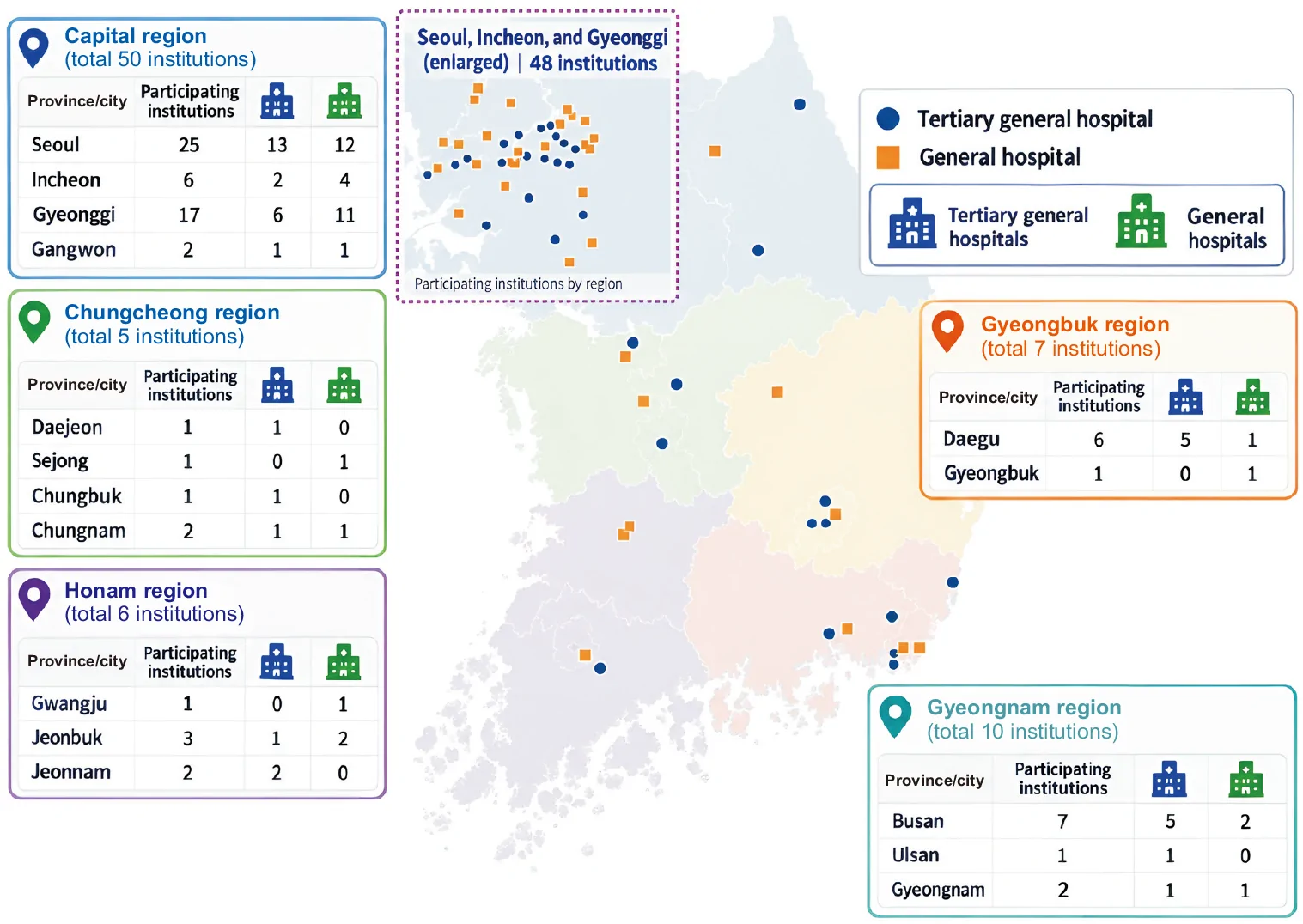
Participation in the first year of the Antimicrobial Steward­ship Program pilot project by healthcare institution’s type and region

**Figure 3:**
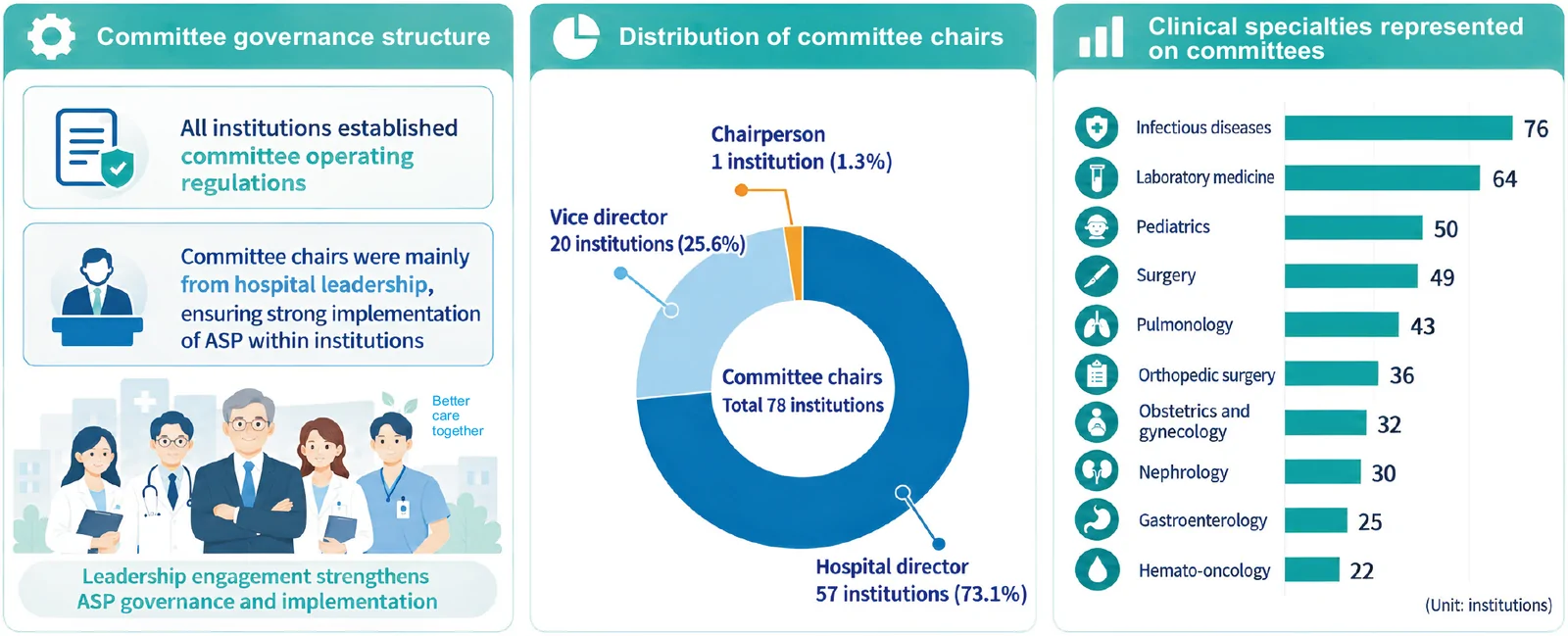
Status of the establishment and operation of an antimicrobial use management committee in participating institutions ASP=Antimicrobial Stewardship Program.

**Table 1:** Assessment criteria for ASP activities for participating institutions

Category	Assessment domain		Assessment item	Maximum score
A. Core criteria	A.1 Team establishment and operation		A.1.1 Establishment and operation of a dedicated ASP team, including completion of training by dedicated personnel A.1.2 Operation of the Antimicrobial Use Management Committee and the reporting of its activities to hospital management	-
	A.2 Guideline development		A.2.1 Development and implementation of guidelines for antimicrobial use	-
	A.3 Antimicrobial use management activities	Interventions	A.3.1 Audit and feedback regarding antimicrobial prescriptions A.3.2 Operation of a restricted antimicrobial approval program	-
		Surveillance	A.3.2 Monitoring of antimicrobial consumption and antimicrobial-resistant pathogens, including the enrollment effects of KONAS A.3.3 Regular surveillance of CDI	-
		Education	A.3.4 Education on antimicrobial prescribing guidelines for physicians on A.3.5 ASP-related education for hospital management and staff	-
	A.4 Outcome reporting		A.4.1 Reporting of ASP activity outcomes	-
B. Additional-score criteria	B.1 Team establishment and operation		B.1.1 Efforts to secure specialized personnel (physicians and pharmacists concentrating in infectious diseases)	60
			B.1.2 Appropriate use of financial support	20
	B.2 Antimicrobial use management activities		B.2.1 Establish an information technology system to support ASP activities	10
			B.2.2 Efforts to implement active antimicrobial stewardship interventions	10

ASP=Antimicrobial Stewardship Program; KONAS=Korea National Antimicrobial Use Analysis System; CDI=*Clostridioides difficile* infection.

**Table 2:** Assessment criteria for ASP grading criteria for participating institutions

Grade	Four grades according to their additional scoresa)			
	A	B	C	D
Additional score (out of 100)	More than 80 points	70–80 points	60 to less than 70 points	Less than 60 points
Financial support	150% of the base funding	130% of the base funding	110% of the base funding	100% of the base funding

ASP=Antimicrobial Stewardship Program. ^a)^Each institution selected for a given year is evaluated based on its ASP activities during that year. All core criteria must be met to qualify for Grade D or higher; institutions are then classified into four grades according to their additional scores.

**Table 3:** Scope of the ASP pilot project and status of ASP personnel

Category	Subcategory	Result
Participating institutions	Total/tertiary general hospitals /general hospitals	78/40/38 institutions
Participation rate	Eligible institutions with >300 beds	45.9% (78/170 institutions)
Regional distribution	Provinces/cities represented:/the metropolitan region (Seoul, Incheon, Gyeonggi, and Gangwon)/non-metropolitan regions	16 provinces/cities/ 64.1% (50/78 institutions)/ 35.9% (28/78 institutions)
Dedicated ASP team	Total participating personnel/mean personnel per institution	695/8.9
Core personnel	Dedicated physicians/dedicated pharmacists and pharmacy support personnel	317/148
Multidisciplinary personnel	Nursing, microbiology, information technology, administration, etc.	230
Specialized personnel	Infectious diseases specialists/infectious diseases specialist pharmacists	234/18

ASP=Antimicrobial Stewardship Program.

**Table 4:** Key operational outcomes in the first year

Domain	Indicator	Result
Guidelines	Antibiotic use guidelines developed	622 guidelines; mean number of guidelines per institution 8.0 (range, 1–43)
Interventions	Mean number of intervention activities performed	5.7
	Most frequently performed intervention/number of institutions	Therapeutic drug monitoring for specific antibiotics/71 (91.0%)
	Least frequently performed intervention/number of institutions	Antibiotic de-escalation/52 (66.7%)
	Average antibiotic prescription ratea)	57.8% (range, 16.0–86.7%)
	Mean institutional acceptance rate/overall intervention acceptance rateb)	81.7%/81.9%
Restricted antibiotics	Mean number of antimicrobial agents managed/Mean approval rate	17.9/89.5%
Antibiotic use	Mean overall antibiotic use per institution	743.0 DOT/1,000 patient-days (range, 273.5–1,297.3)
Education	Guideline education for physicians	Mean, 5.3 sessions
	Awareness education for staff and hospital leadership	Mean, 6.9 sessions
Promotion	Institutions conducting promotional activities/mean number of activities per institution	63 institutions/3.6
Information system	ASP activity support system	73 institutions (93.6%)
Financial support	Pre- and post-implementation financial support	KRW 65.97 billion
Evaluation	Institutions meeting basic indicators/institutions rated A or B	78 (100%)/64 (82.1%)

DOT=days of therapy; ASP=Antimicrobial Stewardship Program. ^a)^Average antibiotic prescription rate: calculated as the average of the antibiotic prescription rates across 78 participating institutions. For each institution, this rate represents the proportion of admitted patients prescribed antibiotics for reasons such as infection or surgery, relative to the total number of monitored patients. ^b)^The mean institution-level intervention acceptance rate gives the average across 78 participating institutions of the proportion of ASP team recommendations to clinicians resulting in prescription changes during the first-year project period. The overall intervention acceptance rate represents the proportion of accepted interventions (263,373) out of all interventions performed by ASP teams (321,661) across the 78 participating institutions during the same period.

## Footnotes

1) DID: A standardized indicator of antibiotic consumption representing the defined daily dose of antibiotics used per 1,000 inhabitants per day (DDD)

2) ASP intervention acceptance rate: Defined as the ratio of the number of cases in which clinicians accepted the intervention and modified the prescription relative to the total number of intervention cases proposed to clinicians by the dedicated ASP team.

3) The restricted antibiotic acceptance rate was defined as the proportion of restricted antibiotic prescription requests submitted through the hospital’s electronic system that were ultimately approved for meeting the dedicated ASP team’s guideline criteria or where the prescribing physician accepted a rejection decision without objection.
