# Supplementary material for: First Year Implementation and Performance of the Republic of Korea’s National Antimicrobial Stewardship Program Pilot: Policy Directions for Sustainable Expansion
Source: Public Health Wkly Rep. 2026 Nov 12;19(43):1802–23. doi: 10.56786/PHWR.2026.19.43.2 (PMC13759228; doi:10.56786/PHWR.2026.19.43.2)
Supplement: Translation

# Translation: 국가 항생제 적정사용 관리 시범사업 1차 연도 운영 성과와 정책 방향

**Authors:** 영일 정, 여원 진, 인경 김, 유진 조, 은영 최, 나리 신

**Affiliations:** 질병관리청 의료안전예방국 항생제내성관리과

**Keywords:** 항생제 적정사용 관리, 항생제 내성, 항생제 사용량, 보건정책

## Abstract

**목적:**

본 보고는 국가 주도의 항생제 적정사용 관리(Antimicrobial Stewardship Program, ASP) 시범사업 1차 연도의 운영 현황과 성과를 분석하고, 향후 사업의 지속적 운영과 의료기관의 단계적 확대를 위한 정책 방향을 제시하고자 하였다.

**방법:**

2024년 11월 1일부터 2025년 12월 31일까지 300병상 초과 종합병원급 이상 78개 참여기관의 사업 수행자료를 분석하였다. 기관별 중간‧연차 결과보고서, 사업지침, 연차평가 및 현장점검 자료를 활용하여 ASP 운영 기반과 주요 구조‧과정지표의 이행 수준을 종합적으로 분석하였다.

**결과:**

전체 대상기관 170개소 중 78개소(45.9%)가 ASP 시범사업에 참여하였다. 참여기관은 총 695명의 다학제 ASP 인력을 구성하였으며, 622종의 기관별 항생제 사용지침을 마련하였다. 처방 중재는 7개 항목 중 기관당 평균 5.7개를 수행하였고, 평균 중재 수용률은 81.7%였다. 제한항생제는 68개 성분을 관리하였으며 기관별 평균 승인율은 89.5%였다. 73개소(93.6%)에서 ASP 활동지원 전산시스템을 운영하였고, 모든 기관이 기본평가항목을 충족하였으며 64개소(82.1%)가 A‧B등급을 받았다. 1차 연도 참여기관에 대한 지원금은 총 659.7억원이었다.

**결론:**

1차 연도 시범사업을 통해 국가 주도의 재정지원과 평가체계를 기반으로 의료기관 내 ASP의 전문인력, 운영체계 및 실행기반을 구축하고 높은 수준의 사업 이행을 확인하였다. 다만 초기 사업의 특성상 항생제 사용량과 내성률 등 결과지표의 단기적 변화를 평가하는 데 한계가 있었다. 향후 종단적 성과평가를 통해 정책효과를 검증하고, 기관별 인력‧인프라 격차를 고려한 맞춤형 지원과 결과 중심의 평가체계로 단계적으로 전환하여 종합병원 및 병원급 의료기관으로 ASP를 확대할 필요가 있다.

**핵심요약**

**① 이전에 알려진 내용은?**

시범사업 이전 국내 의료기관의 항생제 적정사용 관리(Antimicrobial Stewardship Program, ASP)는 기관별 자율적 노력에 의존하여 표준화된 운영체계가 미흡하였으며, 전문인력‧전산 및 재정지원 부족으로 기관별 ASP 수행 역량에 차이가 있었다.

**② 새로이 알게 된 내용은?**

국가 주도의 ASP 시범사업 1차 연도에 78개 기관이 참여하여 695명의 다학제 인력을 구성하고 622종의 항생제 사용지침을 마련하였다. 기관당 평균 5.7개의 중재활동을 수행하였으며, 평균 중재 수용률은 81.7%로 나타났다. 또한 93.6%의 기관에서 ASP 활동지원 전산시스템을 운영하였고, 82.1%가 A‧B등급을 획득하였다.

**③ 시사점은?**

국가 차원의 재정지원과 평가체계는 의료기관의 ASP 운영 기반과 실행력을 강화하는 데 기여할 수 있음을 확인하였다. 향후에는 기관별 인력‧인프라 격차를 고려한 맞춤형 지원과 결과 중심의 평가체계를 마련하고, 종단적 성과평가를 통해 정책효과를 검증하면서 ASP를 단계적으로 확대할 필요가 있다.

## 서 론

항생제 내성은 감염치료의 효과와 의료 안전을 위협하는 주요 공중보건 문제이다. 우리나라의 항생제 사용량과 주요 병원균의 내성률은 특수한 의료적 환경을 고려해도 세계적으로 높은 수준으로 유지되고 있다. 2024년 인체 항생제 사용량은 33.6 DID (defined daily doses per 1,000 inhabitants per day, 인구 1,000명당 1일 사용량)^1)^로 OECD 평균 19.7 DID의 약 1.7배였으며, 2021년 이후 다시 증가하는 양상을 보였다[1-2]. 또한 내성 발생 위험이 상대적으로 낮은 Access 항생제의 사용 비중은 38.8%에 그쳐, 항생제 사용량뿐 아니라 사용 적정성도 개선할 필요가 있다[3]. 내성률 측면에서는 2023년 메티실린내성황색포도알균(methicillin-resistant *Staphylococcus aureus*)이 45.2%, 다제내성 아시네토박터바우마니균(multidrug-resistant *Acinetobacter baumannii*)이 73.7%로 높은 수준을 보였으며, 카바페넴내성 장내세균(carbapenem-resistant *Enterobacteriaceae*)은 지속적인 증가 추세를 나타냈다. 2024년 감시 결과에서도 주요 병원균의 내성률이 전반적으로 높은 수준을 유지하였고, 특히 카바페넴내성 폐렴막대균(carbapenem-resistant *Klebsiella pneumoniae*)은 2016년 이후 급격하게 증가하고 있으며, 요양병원의 내성률이 다른 의료기관보다 높은 것으로 확인되었다[4].

세계보건기구(World Health Organization, WHO)는 항생제 부작용과 불필요한 사용을 줄이고, 치료 효과를 높이는 동시에 항생제 내성균의 발생과 확산을 억제하기 위한 핵심 전략으로 의료기관의 적극적인 항생제 적정사용 관리(Antimicrobial Stewardship Program, ASP) 활동을 권고하고 있다[5]. 효과적인 ASP 수행을 위해서는 경영진의 지원과 책임 있는 전담팀 구성을 기반으로 항생제 사용지침 마련, 제한항생제 사전승인, 전향적 처방 검토와 피드백, 투여기간‧용량‧투여경로의 최적화 등을 시행해야 한다. 또한 항생제 사용량과 내성률을 지속적으로 모니터링하고, 결과 환류와 의료진 교육을 병행하는 체계적인 운영이 필요하다[6].

그간 국내 의료기관의 ASP 활동은 기관별 자율적 노력에 의존하여 체계적인 운영체계와 표준화된 관리기준이 충분히 마련되지 못하였다. 또한 감염내과 전문의와 감염전문약사 등 전문인력 및 전담인력 확보가 제한적이었고, 항생제 처방‧사용량을 체계적으로 모니터링하고 피드백할 수 있는 정보시스템과 평가체계도 미흡하였다. 이에 따라 기관 간 ASP 활동 수준에 상당한 차이가 있었으며, 지속적이고 체계적인 항생제 관리활동을 수행하는 데 제약이 많았다[7-10]. 이에 질병관리청은 보건복지부, 건강보험심사평가원, 국민건강보험공단 및 관련 학회‧전문가들과 함께 국민건강보험재정 기반의 ASP 시범사업 운영계획안을 준비하여, 2024월 9월 4일 제17차 건강보험정책심의위원회를 통해 최종 승인을 얻어 2024년 11월부터 2027년 12월까지 총 3년 2개월에 걸쳐 국가 주도의 ASP 시범사업을 운영하게 되었다[7].

본 보고에서는 국가 주도의 ASP 시범사업 1차 연도의 운영 현황과 성과를 분석하고, 향후 사업의 지속적 운영과 의료기관의 단계적 확대를 위한 정책 방향을 제시하고자 하였다.

## 방 법

본 연구에서는 1차 연도 시범사업에 참여한 300병상 초과 종합병원급 이상 78개 의료기관을 대상으로 2024년 11월 1일부터 2025년 12월 31일까지 수행된 사업 내용을 종합적으로 분석하였다. 특히, 각 의료기관이 제출한 중간 및 연차 결과보고서뿐만 아니라, 사업지침, 연차 결과 평가, 현장점검 자료 등을 적극 활용하였다.

## 결 과

### 1. 항생제 적정사용 관리 시범사업의 개요

본 시범사업의 운영 목적은 의료기관 내 ASP 운영 기반과 상시 이행 체계를 마련하여 항생제를 적절하게 처방‧관리할 수 있도록 유도하는 데 있다(그림 1). 본 사업이 갖는 정책적 의미는 정책–관리평가–재정지원–현장 실행의 유기적 연계를 통해, 사업 이전 의료기관의 자율적 ASP 활동 체계에서 국가 정책과 재정지원을 기반으로 한 체계적 국가사업으로의 전환점이라는 데 있다.

시범사업은 병상 규모에 따른 최소 필수인력 기준을 설정하고, 의료기관별 다학제 ASP 전담팀과 의사결정기구를 구성하도록 설계되었다. 참여기관은 항생제 사용지침 마련, 처방 중재, 제한항생제 승인, 사용량‧내성률 모니터링, 교육‧홍보 및 전산체계 구축 등의 핵심 활동을 성실히 수행해야 하며, 병상 규모와 ASP 활동 수행 결과를 평가하여 A에서 D까지 등급별로 지원금을 차등하여 지원한다(표 1, 2). 사업 참여가 가능한 대상 의료기관은 광범위 항생제 사용량과 입원 및 중증환자가 많아 항생제 내성 관리 시급성과 내성 위험이 높은 300병상 초과 종합병원급 이상부터 우선 적용하였다.

### 2. 1차 연도 참여 의료기관과 전문인력

사업 참여가 가능한 170개 의료기관 중 제주를 제외한 16개 시‧도 소재 78개 의료기관(45.9%)이 사업에 참여하여 사업 원년임에도 의료현장의 높은 관심과 정책적 수요를 확인할 수 있었다. 참여기관 중 수도권(서울‧인천‧경기‧강원) 소재 기관의 참여율이 64.1% (50/78개소)로 가장 높았으며, 종별로는 상급종합병원은 전국 47개소 중 40개소(85.1%), 종합병원은 123개소 중 38개소(30.9%)가 참여하였다. 특히, 중증‧고난도 진료를 담당하는 대부분 상급종합병원의 국가 ASP 관리 체계로의 편입은 향후 ASP의 단계적 확대를 위해 고무적인 부분으로 평가된다(그림 2).

동 사업에 참여한 의료인력은 총 695명으로 기관당 평균 8.9명이 ASP 전담팀을 구성하였다. 이 중 필수인력은 총 465명으로 의사 317명과 약사(약사 지원인력 포함) 148명이 참여하였고, 간호‧임상미생물‧정보기술‧행정 전문가 등을 구성한 다학제 전문인력은 78개 기관에서 230명이 참여하였다. 특히, 참여 의사 중 감염전문의는 234명(73.8%)으로, 국내 감염전문의 10명 중 6명 이상이 참여(64.1%)하여 사업 첫해임에도 높은 ASP 전문성을 확보하였다. 감염전문약사는 14개 기관에서 18명이 참여하여 전체 약사인력의 12.2% 수준이나, 사업 당시 전국 감염전문약사 33명으로 참여 가능 인력이 많지 않은 점을 고려했을 때 54.5%에 해당하는 인력이 사업에 참여하였다(표 3).

### 3. 항생제 사용관리위원회 운영과 항생제 사용지침의 마련

기관 내 성공적인 ASP 운영체계 안착을 위해서는 주요 경영진 중심의 의사결정 체계를 통해 책임 있는 운영과 지원을 기반으로 성과관리–환류–개선으로 이어지는 선순환 구조의 ASP 거버넌스를 확립하여야 한다. 1차 연도 사업기간 내 모든 기관에서 병원장 등 주요 경영진을 위원장으로 하여 항생제 사용관리위원회를 구성하고 운영함으로써 원내 ASP 추진 동력을 확보하였다. 기관당 위원 수는 평균 16.7명이었으며 간호부, 약제부, 행정부 등 간부진과 함께 항생제 처방과 연관된 다양한 진료과(기관 평균 8개 전문 진료과) 의료진 구성으로 다학제 협업 기반을 마련하였다(그림 3). 이로써, ASP 핵심 전략인 다학제‧경영진이 참여하는 정례적 위원회 운영을 통한 의료기관 차원의 지속 가능한 ASP 실행 체계를 마련하였다.

위원회 운영과 함께 각 의료기관의 진료환경과 미생물학적 특성이 반영된 질환별 항생제 사용지침을 마련함으로써 실제 진료 현장에 적용할 수 있는 ASP 기준을 확립해야 한다. 이를 위해 참여기관에서는 학회‧질병관리청‧WHO 발행지침 등을 토대로 기관별 특성을 반영하여 다양한 감염질환에 대한 임상 현장 적용성을 높인 총 622종(기관 평균 8종)의 맞춤형 항생제 적정처방 기준을 마련하였다(표 4). 기관별 최소 1종(5개소)에서 최대 43종(1개소)으로 기관 간 격차는 있었으나, 일관성 있는 항생제 처방 알고리즘으로 단순 처방 기준 제시를 넘어 실제 ASP 중재에 활용 가능한 실무형 관리 체계로 고도화하는 계기가 된 점은 동 사업의 고무적 성과이다.

### 4. 체계적인 항생제 적정사용 관리 선순환 체계 구축

항생제 처방 중재는 의료진의 항생제 처방을 체계적으로 검토하고 적정한 처방으로 유도함으로써 불필요하거나 부적절한 항생제 사용을 줄이고 항생제 내성 발생을 예방하는 ASP의 핵심 활동이다. 이를 통해 실제 처방 변화와 함께 항생제 사용 저감으로 연결하는 실효적인 중재 실행 효과가 의료 현장에서 구현되어야 한다. 본 사업에서는 전향적 감사와 피드백을 중심으로 기관별 여건에 따라 다양한 처방 중재활동을 수행하였다. 사업지침에서 제시하는 7개의 중재항목인 동일성분이나 항균범위가 겹치는 항생제 중복처방을 조정하는 항생제 병합 처방 중재, 최적 투약 기간을 권고하는 장기투여 중재, 약물 부작용과 감염 차단을 위한 주사 항생제의 경구 전환 중재, 미생물 검사 결과와 환자상태에 따라 정밀한 항생제 사용을 권장하는 항생제 하강(antimicrobial de-escalation) 치료 중재, 미생물 배양 및 항생제 감수성 검사 결과를 바탕으로 감염 원인균에 가장 적절한 항생제 사용을 유도하는 미생물 검사 기반의 항생제 처방 중재, 가이드라인에 맞는 항생제 처방 중재 및 환자의 혈중 약물 농도를 실시간 해석하여 환자별 최적 복용량과 투여 간격을 제시하는 특정 항생제에 대한 치료 약물 모니터링이 해당된다. 1차 연도 사업기간 내 참여기관은 7개의 중재항목 중 평균 5.7개의 중재항목에 대해 적극적으로 시행하였다(표 4). 특히 전체 중재건수 대비 처방의가 수용한 평균 중재 수용률^2)^은 81.7%로 나타나, ASP 전담팀의 중재가 실제 임상현장에서 처방 변화로 이어질 수 있음을 확인하였다(표 4). 중재항목별로는 특정 항생제에 대한 치료적 약물 모니터링이 71개 기관으로 가장 높은 수행률을 보인 반면, 항생제 하강 치료는 52개 기관으로 상대적으로 낮았다. 또한 상급종합병원의 중재항목별 수행률이 종합병원보다 전반적으로 높았으며, 특히 가이드라인에 따른 항생제 처방에서 기관 종별 차이가 크게 나타나 기관별 인력과 인프라를 고려한 지원의 필요성을 확인하였다. 일부 기관에서는 사업 이후 전체 항생제 및 광범위‧제한항생제 사용 감소 등 구체적인 성과사례도 확인되었다. 현 시점에서 일부 기관의 성과사례는 사업 전체의 효과로 판단하기 어려우나, 이러한 결과는 ASP가 단순한 관리체계를 넘어 실제 항생제 처방행태와 사용량 변화에 영향을 미칠 수 있음을 보여주며, 향후 중재 수용률과 처방 변경‧중단 등 질적 성과를 지속적으로 평가하여 ASP의 효과를 체계적으로 검증할 필요가 있음을 시사한다.

제한항생제 승인 관리는 광범위하고 내성 위험이 높은 항생제의 불필요하거나 부적절한 사용을 사전에 관리하여 환자에게 적절한 항생제 선택을 유도하고, 항생제 내성 발생을 억제하기 위한 핵심적인 ASP 활동이다. 본 사업에서는 의료기관의 특성과 항생제 내성 위험 등을 고려하여 제한항생제를 지정하고, 처방 전 승인체계를 구축‧운영하도록 하였다. 참여기관은 카바페넴 등을 중심으로 총 26개 계열, 68개 성분을 관리하였으며, 전체 제한항생제 승인 신청 571,197건 중 515,109건이 승인되어 전체 승인율은 90.2%로 나타났다. 기관별 평균 승인율은 89.5%, 중앙값은 91.4%였으며 기관별로 42.9–100%의 차이를 보였다(표 4). 이러한 차이는 ASP 도입 시기와 운영의 정착 정도, 처방의사의 인식 및 기관 내 협업 수준 등에 영향을 받을 수 있어 기관 간 단순 비교보다는 기관별 지속적인 모니터링이 중요하다. 특히 일부 기관에서는 승인체계의 적극적인 운영을 통해 제한항생제 승인율^3)^과 사용량이 감소하는 등 처방행태의 개선이 확인되는 등 제한항생제 승인 관리 사례에서 승인율이 14.8–16.7% 감소하였고, 일부 기관에서는 meropenem, vancomycin, teicoplanin 등의 사용량도 감소하였다. 이는 제한항생제에 대한 사전 관리체계가 실제 항생제 처방 및 사용행태 개선으로 연결될 가능성을 보여주는 결과로, 지속적인 사용량‧처방행태 모니터링을 통해 ASP의 효과를 장기적으로 평가할 필요가 있다.

이 밖에도, 사업을 통해 표준화된 내성균‧클로스트리디오이데스 디피실 감염증(*Clostridioides difficile* infection, CDI) 발생 감시체계 구축을 통해 항생제 사용량‧중재활동 등 주요 결과지표와 연계 분석이 가능한 체계를 마련하였으며, 내성 부담이 높은 균종과 의료기관 특성을 파악하여 향후 중점 관리에 필요한 정책적 근거 마련을 위한 기반을 확보하였다.

### 5. 의료진 처방행태 변화와 항생제 내성 인식개선을 위한 항생제 적정사용 관리 교육 활동

ASP 교육‧홍보 활동은 의료진의 항생제 사용지침 준수와 적정 처방을 촉진하고, 경영진 및 직원의 항생제 내성 및 ASP에 대한 인식을 높여 의료기관 내 ASP가 지속적으로 운영될 수 있는 기반을 마련하는 데 목적이 있다. 본 사업에서는 처방의를 대상으로 한 항생제 사용지침 교육과 직원‧경영진 대상 ASP 인식 개선 교육을 병행하여 기관 전반의 참여와 협업을 유도하였다. 모든 참여기관에서 지침 교육을 시행하였으며, 기관당 평균 5.3회의 교육이 운영되었다. 또한 직원‧경영진 대상 인식교육은 모든 기관에서 시행되었고 기관당 평균 6.9회로, 처방의 대상 지침 교육보다 높은 빈도로 이루어졌다(표 4). 사업 이행의 필수 사항이 아니었던 홍보활동도 63개소(80.8%)에서 자발적으로 수행되었으며, 기관당 평균 3.6회의 활동이 이루어져 ASP에 대한 기관 내외의 인식 확산을 위한 노력이 확인되었다(표 4). 특히 일부 기관에서는 지침 개발 이후 처방의 대상 교육과 만족도 조사를 통해 중재 저해 요인을 파악‧개선한 결과, 7개 중재항목의 평균 수용률이 57.5%에서 92.9%로 향상되었다. 또한 병원장이 직접 ASP 교육에 참여한 기관에서는 중재 수용률이 평균 94%로 높게 나타났다. 이러한 결과는 ASP 교육‧홍보가 단순한 정보 전달을 넘어 의료진의 처방행태와 중재 수용성 향상에 기여할 수 있음을 보여주는 결과이다.

### 6. 참여의료기관 평가 및 지원금

1차 연도 참여기관 평가 결과, 모든 기관이 기본항목을 충족하였으며 64개소(82.1%)가 A‧B등급으로 평가되어 전반적으로 높은 사업 이행 수준과 참여 의지를 확인하였다(표 4). 특히 301–600병상 기관에서도 D등급 없이 86.7%가 A‧B등급을 획득하여 상대적으로 제한된 인프라에도 불구하고 적극적인 ASP 활동을 수행한 것으로 나타났다. 반면 기관 간 등급 차이는 주로 감염전문의와 약사 등 전문인력 확보 수준에서 발생하였다. 이는 ASP의 안정적 운영을 위해 기관별 인력과 인프라 여건을 고려한 맞춤형 지원과 함께 전문인력 확보를 강화하는 정책적 지원이 필요할 것이다. 평가 결과 반영된 1차 연도 참여기관 지원금 총액은 659.7억원으로 사전지원금 약 246억 원과 사후지원금 약 414억 원이 지급되었다(표 4).

## 결 론

건강보험 수가 기반의 국가 주도 의료기관 ASP 지원 정책을 통해 그간 인력과 재정적 제약으로 자체적인 ASP 운영이 어려웠던 의료기관에 체계적인 관리체계를 도입하고, 항생제 사용의 질 향상과 환자 안전 확보 및 항생제 내성 발생‧확산 억제를 위한 국가 차원의 관리 기반을 마련하였다는 점은 본 시범사업의 중요한 첫해 성과라 할 수 있다.

1차 연도 사업을 통해 참여기관에서는 기본지표를 충족하고 위원회 운영, 사용지침 마련, 처방 중재, 감시 및 교육을 중심으로 상시적인 ASP 운영체계를 구축함으로써 높은 사업 이행 수준과 현장 정착 가능성을 보여주었다. 특히, 감염전문의를 비롯한 전문인력과 다학제 인력의 참여는 전문성과 협업에 기반한 처방 관리체계 구축에 기여하였으며, 64.1%의 감염전문의 참여율은 ASP의 초기 도입과 정착에 있어 주요성과로 평가된다. 향후 감염전문의는 선도기관과 도입기관 간 협업 네트워크를 통한 ASP 확산에도 중추적 역할을 수행할 것으로 기대된다. 또한 약사는 ASP 중재활동을 실질적으로 수행하는 핵심인력으로서 그 역할과 중요성이 확인되었으며, 사업 확대에 대비한 전문약사 인력의 지속적인 양성이 필요하다.

중재활동 측면에서도 다양한 중재항목이 단기간에 도입‧운영되었으며, 기관 평균 중재 수용률은 81.7%로 나타나 ASP 전담팀의 중재활동이 실제 임상현장에서 수용되고 있음을 확인하였다. 다만, 항생제 병합(중복) 처방 중재 등 일부 항목의 수용률은 62.5%로 상대적으로 낮아, 중재의 효과성을 높이기 위한 경영진의 지원과 다학제 협력 강화가 필요하다. 아울러 항생제 사용량, 내성률 및 CDI 발생 현황에 대한 지속적인 감시체계를 마련함으로써 향후 항생제 처방과 사용량, 내성 변화 간의 연관성을 종합적으로 평가할 수 있는 기반을 확보하였다.

앞서 언급했듯이 본 사업은 국내 의료기관을 대상으로 ASP의 구조적 기반을 마련함으로써 기관별 여건에 따른 다양한 중재활동을 도입‧정착시켜 향후 지속적인 ASP와 사업 확대를 위한 기반을 확보하였다는 점에서 의의가 있다. 일부 참여기관에서 전체 항생제 및 광범위‧제한항생제 사용량 감소, 교육에 따른 중재 수용률 증가 등의 성과가 확인되었으나, 이를 전체 참여기관의 공통적인 효과로 일반화하기는 어렵다. 따라서 향후에는 3개년 시범사업 기간의 누적 자료를 활용하여 의료기관의 특성과 ASP 운영 수준에 따른 중재활동의 효과를 다각적으로 분석할 필요가 있다. 구체적으로 상급종합병원과 종합병원 등 의료기관 종별, 감염내과 설치 및 감염전문의 참여 여부, 전담인력의 규모‧구성‧투입 수준에 따른 중재 수행량과 수용률, 항생제 처방 변화 등을 비교‧평가해야 한다. 또한 의료기관별 전산시스템의 구축‧운영 유형과 처방 감시, 중재 지원, 사용량 자동 산출 등 주요 기능의 활용 수준을 분석하여 전산시스템이 ASP 운영 효율성과 중재 성과에 미치는 기여도를 평가할 필요가 있다. 나아가 항생제 사용량과 주요 내성균의 내성률 등에 대한 종단적 변화를 추적하고, 기관별 기초 특성과 사업 참여 이전의 수준을 고려한 비교분석을 통해 ASP 시행에 따른 중장기 효과를 객관적으로 검증해야 할 것이다.

궁극적으로 이러한 분석 결과는 의료기관별 ASP 운영 성과의 차이와 주요 영향요인을 규명하고, 효과적인 인력 배치와 전산시스템 활용 전략 등 근거 기반의 사업 운영모형을 마련하는 데 활용될 수 있을 것이다. 또한 국내 의료기관의 지속가능한 ASP 운영과 전체 종합병원으로의 단계적 확대를 위해서는 시범사업의 본사업 전환을 추진하는 한편, 의료기관의 규모와 전문인력‧인프라 수준을 고려한 차별화된 지원 및 선도기관 중심의 협업체계를 마련하여 ASP 도입 경험이 부족한 의료기관의 조기 정착을 지원할 필요가 있다.

## Figures and Tables

**그림 1:** 항생제 적정사용 관리(Antimicrobial Stewardship Pro­gram, ASP) 운영 기반 및 상시 이행 체계

**그림 2:** 항생제 적정사용 관리 시범사업 1차 연도 종별 및 권역별 참여의료기관 현황

**그림 3:** 참여의료기관의 항생제 사용 관리위원회 구성 및 운영 현황 ASP=Antimicrobial Stewardship Program.

**표 1:** 사업 참여기관의 항생제 적정사용 관리(Antimicrobial Stewardship Program, ASP) 활동 수행 평가 항목

구분	평가 영역		주요 ASP 활동 수행 평가 항목	배점
A 기본 항목	A.1 전담팀 구성‧운영		A.1.1 ASP 전담팀 구성‧운영(전담인력 교육이수 포함) A.1.2 항생제 사용관리위원회 운영 및 운영 결과의 경영진 보고	-
	A.2 지침 마련		A.2.1 항생제 사용지침 마련‧적용	-
	A.3 항생제 사용 관리활동	중재활동	A.3.1 항생제 처방에 대한 감사와 피드백 A.3.2 제한 항생제 승인 프로그램 운영	-
		추적조사	A.3.3 항생제 사용량 및 항생제 내성균 현황 감시(KONAS 가입 여부) A.3.4 CDI 감염증 정기 추적 관찰	-
		교육활동	A.3.5 항생제 처방 지침 교육(의사 대상) A.3.6 ASP에 관한 교육(경영진과 직원 대상)	-
	A.4 결과 보고		A.4.1 ASP 활동 결과에 대한 보고	-
B 가산 항목	B.1 전담팀 구성‧운영		B.1.1 전문인력(감염전문의, 약사) 확보 노력	60점
			B.1.2 지원금 적정 집행 노력	20점
	B.2 항생제 사용 관리활동		B.2.1 ASP 활동에 필요한 전산시스템 구축	10점
			B.2.2 적극적인 중재활동 이행 노력	10점

KONAS=Korea National Antimicrobial Use Analysis System; CDI=*Clostridioides difficile* infection.

**표 2:** 사업 참여기관의 항생제 적정사용 관리(Antimicrobial Stewardship Program, ASP) 평가 등급 산정 기준

평가등급	가산 점수에 따른 평가 등급 산정 기준a)			
	A등급	B등급	C등급	D등급
가산점수(100점 만점)	80점 초과	70점 이상–80점 이하	60점 이상–70점 미만	60점 미만
지원금 지급기준	기본지원금의 150%	기본지원금의 130%	기본지원금의 110%	기본지원금의 100%

a) 매 연차 선정된 의료기관에 대해 해당 연차 ASP 활동 결과를 평가하여 기본항목을 모두 충족(D 등급)하고, 가산항목 기준에 따라 4개 등급으로 구분하여 평가.

**표 3:** 시범사업 참여범위와 항생제 적정사용 관리(Antimicrobial Stewardship Program, ASP) 인력 현황

구분	세부항목	결과
참여기관	전체/상급종합/종합	78/40/38개소
참여율	300병상 초과 대상기관	45.9% (78/170개소)
지역분포	참여기관 소재 시‧도/수도권(서울‧인천‧경기‧강원) 소재/수도권 외 소재	16개 시도/64.1% (50/78개소)/35.9 (28/78개소)
ASP 전담팀	전체 참여 인력/기관 평균 인력	695명/기관 평균 8.9명
필수인력	전담의사/전담약사 및 약사지원인력	317명/148명
다학제 인력	간호‧미생물‧정보기술‧행정 등	230명
전문인력	감염전문의/감염전문약사	234명/18명

**표 4:** 1차 연도 핵심 운영성과

영역	지표	결과
지침	항생제 사용지침 마련	전체 622종; 기관 평균 8.0종(범위 1종–43종)
중재	수행 항목	평균 5.7개
	기관에서 가장 많이 수행한 항목/수행한 기관수	특정 항생제에 대한 치료 약물 모니터링/71개소(91.0%)
	기관에서 가장 적게 수행한 항목/수행한 기관수(비율)	항생제 하강(antimicrobial de-escalation) 치료/52개소(66.7%)
	기관 평균 항생제 처방률a)	57.8%(범위 16.0–86.7%)
	기관별 평균/통합 중재 수용률b)	81.7%/81.9%
제한항생제	관리 성분 수/기관 평균 승인율	평균 17.9종/89.5%
사용량	기관 평균 전체 항생제 사용량	743.0 DOT/1,000 재원일수(범위 273.5–1,297.3)
교육	의사 대상 지침교육	평균 5.3회
	직원‧경영진 대상 인식교육	평균 6.9회
홍보	시행기관 수/기관 평균 홍보횟수	63개소/3.6회
전산	ASP 활동지원 프로그램	73개소(93.6%)
재정	사전‧사후 지원금	659.7억 원
평가	기본지표 충족/A‧B등급	78개소(100%)/64개소(82.1%)

DOT=days of therapy; ASP=Antimicrobial Stewardship Program. ^a)^항생제 처방률은 1차 연도 사업 기간 내 각각의 참여기관에서 모니터링한 입원환자 수 대비 감염이나 수술 등의 이유로 항생제를 처방받은 환자 수 의 비율을 의미하며, 기관 평균 항생제 처방률은 78개 기관별 항생제 처방률에 대한 평균값을 의미함. ^b)^기관 평균 중재 수용률은 1차 연도 사업 기간 내 각 참여기관의 ASP 전담팀이 임상의에게 제안한 총 종재 건수 대비 임상의가 이를 받아들여 처방을 변경한 건수의 비율에 대한 78개소의 평균값을 의미하고, 통합 중재 수용률은 1차 연도 사업 기간 내 78개 참여기관이 ASP 전담팀이 중재한 중재 건수 총합(321,661건) 대비 총 수용 건수(263,373건)의 비율을 의미함.

## Footnotes

1) DID: 인구 1,000명당 하루 동안 사용된 항생제의 정의일일용량(DDD, defined daily dose)을 나타내는 표준화된 항생제 사용량지표

2) 중재 수용률(definition of ASP intervention acceptance rate): ASP 전담팀이 임상의에게 제안한 총 종재 건수 대비 임상의가 이를 받아들여 처방을 변경한 건수의 비율로 정의하였다.

3) 제한 항생제 수용률은 원내 전산시스템을 통해 신청된 전체 제한 항생제 처방 요청 건수 중, ASP 전담팀의 가이드라인 기준을 충족하여 최종 승인되었거나 승인 거부 처분에 대해 처방 의사가 이의 없이 수용한 건수의 비율로 정의하였다.
